# Development and validation of the IFN-γ FlowCytoScreen for cytokine-based potency assessment of advanced therapy medicinal products

**DOI:** 10.3389/fimmu.2026.1959178

**Published:** 2026-10-20

**Authors:** Silvia Carloni, Claudia Piccinini, Chiara Arienti, Dario Armando Leone, Elena Pancisi, Francesca Fanini, Sara Pignatta, Valentina Soldati, Monica Stefanelli, Anna Maria Granato, Flavia Foca, Jenny Bulgarelli, Laura Ridolfi, Massimiliano Petrini

**Affiliations:** 1 Immuno-Gene Therapy Factory, IRCCS Istituto Romagnolo per lo Studio dei Tumori (IRST) “Dino Amadori”, Meldola, Italy; 2 Miltenyi Biotec s.r.l., Bologna, Italy; 3 Unit of Biostatistics and Clinical Trials, IRCCS Istituto Romagnolo per lo Studio dei Tumori (IRST) “Dino Amadori”, Meldola, Italy; 4 Advanced Cellular Therapies and Rare Tumors Unit, IRCCS Istituto Romagnolo per lo Studio dei Tumori (IRST) “Dino Amadori”, Meldola, Italy

**Keywords:** ATMP, cytokine secretion, ELISpot assay, IFN-gamma, potency

## Abstract

**Introduction:**

Robust quality control methods are required to ensure the safety, efficacy and consistency of advanced therapy medicinal products. Potency assays play a critical role in evaluating their biological activity. While ELISpot remains the reference method for single-cell cytokine analysis, it has several limitations such as a lack of standardisation and limited capacity for multiparametric characterisation. This study aimed to develop and validate a flow cytometry-based potency assay for the assessment of single-cell IFN-γ secretion.

**Methods:**

The commercially available Miltenyi Biotec IFN-γ Secretion Assay - Detection Kit, based on flow cytometry, was modified to assess the potency of dendritic cell-based vaccines. To ensure its reliability, our assay was rigorously evaluated for specificity, linearity, accuracy, robustness and precision. Comparative analysis with the ELISpot assay was also performed to assess agreement between the two methods.

**Results:**

Method development resulted in reduced sample requirements and optimisation of cytokine secretion conditions to generate a response to antigen-presenting cell stimulus. Validation showed that the specificity, linearity, accuracy, robustness and precision consistently met the acceptance criteria. Comparative analysis with ELISpot demonstrated high correlation and agreement, with no evidence of systematic or proportional error.

**Discussion:**

The developed assay provides a reliable, scalable and efficient flow cytometry-based approach for cytokine secretion analysis and represents an alternative method to ELISpot. Its advantages in sensitivity, efficiency and cost effectiveness make it a valuable tool for advanced therapy quality control and immune monitoring in clinical research. The flow cytometry further enhances its potential by enabling multiparametric immune profiling, supporting comprehensive characterisation of cellular products in both preclinical and clinical applications.

## 1 Introduction

The functional assessment of immune cells is fundamental in both biomedical research and clinical applications, particularly in the development and quality control of advanced therapy medicinal products (ATMPs). Among these, cell-based immunotherapies rely on precise potency evaluation to demonstrate biological activity, ensure batch-to-batch consistency, and comply with regulatory requirements (1, 2). However, cellular immunity assays often present significant challenges, including labor-intensive protocols, inherent biological variability, and limited reproducibility compared to other immunological tests. These factors highlight the need for rigorous method validation to guarantee the reliability and standardisation of results, which is essential for clinical translation and regulatory approval (3–5). Currently, the Enzyme-Linked Immunospot (ELISpot) assay is widely recognised as the gold standard for assessing cellular immune responses (6). This method allows for the highly sensitive quantification of cytokine-secreting cells upon specific stimulation, offering valuable insights into immune functionality. Despite its widespread use, ELISpot has inherent limitations, including high cost, technical difficulties, long processing times and a challenging standardisation across operators and laboratories (7, 8). In particular, one of the most critical aspects of ELISpot analysis is the accurate reading and interpretation of results, which can introduce variability and impact data reliability (9, 10). These constraints may reduce its suitability for routine potency testing in regulated manufacturing environments, where reliability, reproducibility, and assay standardisation are essential. To address these limitations, the development of alternative assays that can complement or enhance current methods is an active area of research, particularly in the context of immunotherapy (6, 11–14). In our cell factory, we routinely produce autologous dendritic cell (DC)-based vaccines loaded with tumor homogenate. Two key mechanisms of action relevant to DCs are their co-stimulatory activity and antigen-presenting function (15–17). In particular, interferon (IFN)-γ production was selected as a functional attribute indicating potency because it represents a key downstream readout of T-cell activation, which is central to the proposed mechanism of action of the DC-based vaccine. The biological relevance of this endpoint is supported by previous clinical studies showing associations between vaccine-induced tumor-specific IFN-γ responses and clinical outcomes in patients receiving DC-based vaccines (1, 18). Accordingly, we employed the validated Co-Flow DC Assay (19) and the IFN-γ ELISpot assay as complementary potency assays to evaluate DC co-stimulatory activity and T-cell responses, respectively, in accordance with international guidelines recommending the integration of multiple functional assays for comprehensive immunological product characterisation (4). Although both assays rely on DC-mediated T-cell stimulation, they provide distinct functional readouts: the Co-Flow DC Assay measures T-cell proliferation, whereas the IFN-γ ELISpot assay measures the capacity of stimulated T cells to produce IFN-γ. To streamline this workflow and improve assay standardisation, we developed a flow cytometry-based potency assay as an alternative to the IFN-γ ELISpot. The method was designed to maintain the same overall stimulation approach as the ELISpot assay, while using assay-specific optimised conditions suitable for the flow cytometry-based format. The method also leverages the robustness, multiplexing capability, and widespread availability of flow cytometry platforms (20). Specifically, we developed and optimised a novel flow cytometry-based assay using a commercially available kit for cytokine production analysis in stimulated cells. Following assay optimisation, a comprehensive validation was performed to demonstrate compliance with predefined acceptance criteria for specificity, linearity, accuracy, robustness, and precision. Finally, the reliability of the newly developed assay was assessed through an inter-assay comparability analysis to determine the statistical correlation between its results and those obtained with the IFN-γ ELISpot assay. This protocol provides a standardised, reproducible approach to measuring DC-induced T-cell IFN-γ responses, offering a practical alternative to ELISpot for the potency testing of DC-based vaccines. Furthermore, this workflow can be adapted for use with other ATMPs and immunotherapy settings, where a rapid, reproducible and regulatory-compliant assessment of cellular immune function is required.

## 2 Materials and methods

All the reagents, kits, software and instruments used in this study, along with their respective catalogue numbers, RRID and manufacturers, are listed in **Supplementary Table 1**.

### 2.1 Ethics statement

The DC samples analysed in this study were obtained during the manufacturing process, as detailed in the sections 2.5, within the context of the following clinical trials (Eudract Number): 2012-001410-41, 2014-005123-27, 2015-000894-11, 2019-000793-27, 2020-003755-15, 2024-514053-30-00. The reported data derived from routine quality control testing of the manufactured cell products. Each clinical trial was approved by the Italian National Ethics Committee for ATMPs (CEN ATMP). Fully anonymised peripheral blood samples were supplied by the Transfusion Unit of Pievesestina (Cesena, Italy) from healthy donors who had provided written informed consent at the time of donation. These samples, which consisted of residual material from routine blood donations, were used in accordance with the Declaration of Helsinki, national regulations and institutional policies.

### 2.2 Peripheral blood mononuclear cells (PBMCs) purification and subset cell isolation

Healthy donor blood samples were used to obtain PBMCs by density gradient centrifugation using Lymphocyte Separation Media to select CD3+ T cells and CD14+ monocytes. These cells were obtained by negative depletion using the Pan T Cell Isolation Kit and by positive selection with CD14 MicroBeads, respectively, according to the manufacturer’s recommended protocol. CD3+ T cells and CD14+ monocytes were cryopreserved in 90% heat-inactivated human serum AB and 10% dimethyl sulfoxide (DMSO) solution until use.

### 2.3 Immunophenotypic analysis

To assess the purity of magnetically isolated cell populations, T cells were stained with anti-human Viogreen CD3 recombinant antibody (REA) (1:50), while monocytes were stained with anti-human Viogreen CD14 REA (1:50) for 10 minutes (min) at 4°C in the dark. Conjugated REA Controls (S) (1:50) were included for each sample. Cells were then washed twice in autoMACS running buffer before flow cytometric analysis. Dead cells were excluded from the analysis using 7-amino-actinomycin D (7-AAD) staining solution (1:10) according to the manufacturer’s instructions. Purity of cell samples used for subsequent experiments was always higher than 90%.

### 2.4 Cell culture

The human CD19+ REH cell line was purchased from the American Type Culture Collection (Rockville, MD, RRID : CVCL_1650) and maintained at 37°C and 5% CO_2_ in RPMI supplemented with 10% foetal bovine serum (FBS). Cells were screened for mycoplasma contamination using the MycoAlert Mycoplasma Detection Kit according to the manufacturer’s instructions. The monoclonal anti-human CD19 (1:50) antibody was used to identify the REH cells during IFN-γ secretion experiments.

### 2.5 Generation of Monocyte-derived DCs (Mo-DCs)

Mo-DCs were generated from PBMCs obtained from patients according to a previously described protocol (21). Briefly, PBMCs were cultured in CellGenix GMP DC Medium at 10 × 10^6^ cells/mL for 2 hours (h), after which non-adherent cells were discarded. Adherent cells were further cultured in CellGenix GMP DC Medium supplemented with 1000 IU/mL of recombinant human (rh) Interleukin (IL)-4 and rh-Granulocyte-Macrophage Colony-Stimulating Factor. On day 6, 90% of the DC culture was pulsed with autologous tumor homogenate (100 mg/mL), while the remaining 10% was pulsed with Immucothel. On day 7, the culture medium was replaced with CellGenix GMP DC Medium containing IL-6 (2000 IU/mL), Tumor Necrosis Factor-α (20 ng/mL), IL-1β (20 ng/mL), and Prostaglandin E2 (1 μg/mL). On day 9, the mature dendritic cells were harvested, washed, resuspended in saline solution and counted to assess their viability and purity. The cells were then cryopreserved in 90% autologous plasma and 10% DMSO until use.

### 2.6 Biological sample handling

All samples were subjected to the following steps prior to the assays. Samples were thawed at 37°C, washed, and resuspended in saline solution. A total of 2 × 10^5^ cells from each sample were incubated with Annexin V-FITC in binding buffer (1:40) for 15 min at 37°C in a humidified, dark environment. After incubation, the cells were washed and resuspended in binding buffer. Propidium iodide (PI) was added (1:20) immediately before flow cytometric analysis to distinguish between total apoptotic cells (Annexin V+ and PI− or +) and necrotic cells (Annexin V− and PI+).

### 2.7 IFN-γ secretion assay

A total of 1x10^6^ of viable (Annexin V- and PI-) T cells were resuspended in 90 μl of cold AIM-V medium and incubated with 10 μl of a bi-specific anti-IFN-γ (mouse IgG1)/CD45 (mouse IgG2a) monoclonal antibody (Ab) (Catch Reagent, 1:10 dilution) for 5 min at 4°C. Subsequently, 900 μl of AIM-V medium pre-warmed at 37°C was added.

Viable T cells and DCs were co-cultured at different cell ratios in AIM-V medium in 96-well plates under the following experimental conditions: (i) no-DC control, consisting of T cells stimulated with OKT3 (0.01 µg/mL); (ii) positive control, consisting of T cells, DCs and Cytostim (4 μl/well) added during the last 2 h of culture; (iii) background, consisting of T cells and DCs cultured in the absence of stimulatory reagents; and (iv) COSTIM, consisting of T cells, DCs and OKT3 (0.01 µg/mL). Cells were incubated for 22 h at 37° C in a humidified atmosphere containing 5% CO_2_.

At the end of the incubation period, cells were centrifuged at 1400 rpm for 5 min at 4°C and resuspended in 90 μl of cold buffer (0.5% of BSA and 2 mM of EDTA in PBS 1X). Subsequently, 10 μl of PE-conjugated anti-IFN-γ monoclonal Ab (Detection Ab, 1:10 dilution) was added, and samples were incubated for 10 min at 4°C. Cells were then washed with cold buffer and analysed by flow cytometry. To evaluate assay specificity, the monoclonal anti-human CD54 (1:10), CD86 (1:50), CD80 (1:10) and REA Control (S) Ab, were added to the COSTIM condition.

### 2.8 IFN-γ ELISpot assay

The IFN-γ ELISpot assay was performed according to the MIATA guidelines. A 96-well plate equipped with PVDF membranes was incubated with 20 μL of 70% ethanol for 2 min at room temperature (RT). Anti-IFN-γ Ab (1:100) was then added to each well and incubated overnight (O/N) in the dark at 4°C. Blocking solution was added to each well and incubated for 1h at 37°C in the dark. Subsequently, 1 x 10^5^ viable (Annexin V- and PI-) T cells were seeded in quadruplicate in CellGenix GMP DC Medium under the following experimental conditions: (i) positive control, with 1 µg/mL of phorbol 12-myristate 13-acetate (PMA); (ii) no-DC control, with 0.005 µg/mL OKT3; (iii) background, with 5 x 10^3^ DC (1:20 ratio); (iv) COSTIM, with 5 x 10^3^ DC in the presence of 0.005 µg/mL OKT3. The plate was then incubated for 22 h at 37° C in a humidified atmosphere. Secondary biotinylated Ab (1:100) was added to each well and incubated for 1 h at 37°C in the dark. HRP-conjugated streptavidin was then added to each well and incubated for a further hour at 37°C in the dark. Thereafter, the substrate, diluted in ethanol 30%, and AEC solution were added to each well and incubated for 25 min at RT. Each step was followed by a thorough wash with PBS 1X. The PVDF membranes were then dried at RT in the dark and the results were evaluated using the ELISpot Reader and software. Multiple preliminary determinations performed on different healthy donors, using both positive and no-DC controls, allowed the application of standard spot-reading parameters (Region of Interest = 60%, Minimum Spot Size = 2, Maximum Spot Size = 50,000, Minimum Spot Intensity = 5, Minimum Spot Circularity = 10, Spot Separation = 200, and Color Range = 0-360).

### 2.9 Multiparametric flow cytometry

Multiparametric flow cytometry analysis was performed on samples from the COSTIM experimental condition. Cells were stained with anti-human VioBlue CD4 REA (1:50), VioBright FITC CD137 REA (1:50), PE Detection Ab (1:10), PerCP-eFluor710 CD56 mouse (1:20), PE-Vio770 CD103 REA (1:50), APC CD3 mouse (1:20) and APC-Vio770 CD8 REA (1:50) for 10 min at 4°C in the dark. Conjugated REA and isotype controls were included for each sample. Cells were then washed twice in autoMACS running buffer before flow cytometric analysis. Multiparametric analysis was performed following generation of the compensation matrix using Cytostim-stimulated samples. The compensation matrix was subsequently verified by confirming that the frequency of IFN-γ+ cells measured in the single-stained sample was consistent with that observed in the corresponding sample within the full staining panel.

### 2.10 Flow cytometry

Flow cytometry was performed using the MACSQuant Analyzer 10, equipped with 405 nm (violet), 488 nm (blue) and 640 nm (red) lasers. A total of 10,000 events were recorded per sample. Acquisition and analysis gates were defined based on forward (FSC) and side scatter (SSC) characteristics to identify lymphocytes, monocytes, or DCs. Both FSC and SSC were measured on a linear scale. Data were analysed using the MACSQuantify 2.13.2 software.

### 2.11 Statistical analysis

Potency was calculated by subtracting the percentage of IFN-γ+ cells of the background from the value of the COSTIM condition. Assays were considered evaluable when the background value was between the positive and the no-DC control values. The fold change was calculated as the ratio between the mean value of the DC potency and that of the monocyte potency. The variability of the potency results obtained throughout the method validation was evaluated by applying the percentage coefficient of variation (CV) or the recovery. Linear regression analysis was used to assess the relationship between the number of T cells and IFN-γ potency separately in T cells and REH cells, with the coefficient of determination (R^2^) and the corresponding p-value reported. The difference in potency between T cells and REH cells across the matched experimental conditions was assessed using the paired standardized mean difference (Cohen’s d) (22). Statistical significance was defined as a two-sided p-value <0.05. The integrated mean fluorescence intensity (iMFI) was calculated by multiplying the frequency by the mean fluorescence intensity (MFI) of IFN-γ+ cells (23). To account for the different measurement scales of the two assays, SFC and iMFI values were standardised separately using z-score transformation, calculated as (observed value − mean)/standard deviation. No positivity cut-off was applied for the comparative analysis, and measurements were analysed as continuous variables. Agreement between the two quantitative assays was assessed using Bland–Altman analysis. In this analysis, each pair of standardised SFC and iMFI measurements was represented by its mean, calculated as (std SFC + std iMFI)/2, and its difference, calculated as std SFC − std iMFI. The mean of the paired standardised measurements was plotted on the x-axis to represent the magnitude of the measurements on a common, dimensionless scale, while the difference was plotted on the y-axis to assess systematic bias. The 95% limits of agreement were calculated as the mean difference ± 1.96 standard deviations of the differences. Intraclass correlation (ICC) was calculated as an index of reliability. ICC estimates and their 95% confidence intervals (CI) were calculated based on a mean score, consistency of agreement, and a two-way mixed-effects model (number of groups = 2). ICC values of less than 0.5 indicate poor reliability; values between 0.5 and 0.75 indicate moderate reliability; values between 0.75 and 0.90 indicate good reliability; and values greater than 0.90 indicate excellent reliability (24). Stata/SE version 15.1 for Windows was used for all statistical analyses presented in the paper.

**Table 1** provides a comprehensive summary of the evaluated validation parameters and their corresponding acceptance criteria. Additionally, it shows the number of independent biological samples and technical replicates, representing independently generated biological samples and repeated measurements performed on the same sample, respectively.

**Table 1:** Predefined validation parameters, acceptance criteria and experimental design.

Validation parameter	Acceptance criteria	Indipendent biological sample (n)	Technical replicates (n)
Specificity (biological response)	Percentage variation > 70%	4	1
Specificity (stimulation)	% IFN-γ+ COSTIM+Abs < background	3	1
Specificity (analytical)	Cohen’s d > 0.8	1	1
Linearity	R^2^ ≥ 0.97	3	1
Accuracy	Recovery = 80-120%	3	1
Robustness	CV < 10%	1	1
Repeatability	CV < 10%	1	3
Precision (inter-assay)	CV ≤ 20%	1	3
Precision (inter-day)	CV ≤ 20%	1	3
Precision (inter-analyst)	ICC > 0.5	1	3

### 2.12 Assay and cost comparison

For the comparison of the two assays, matched aliquots obtained from the same cell preparation were used for both methodologies. Sample identity was blinded during assay performance and data analysis.

A cost comparison was performed between ELISpot and IFN-γ FlowCytoScreen. The analysis considered the reagents and consumables required to assess the potency of one DC batch. Sample handling and isolation procedures were excluded from the analysis because they were identical for both assays. Instrument acquisition and maintenance costs were not included in the comparison. For each assay, the cost per well (€/well) was calculated based on the purchase price and the amount of reagent or consumable required per well, and the total material cost per assay (€/assay) was then calculated according to the number of wells required. The analysis also considered the operator time required to perform each assay, reported as hands-on laboratory time and total assay duration. All costs were calculated based on the purchase prices applicable at the time of the analysis.

## 3 Results

### 3.1 Method optimisation

IFN-γ secretion was used as a functional readout of DC-induced T-cell activation following co-culture with CD3+ T lymphocytes and a suboptimal dose of anti-CD3 Ab (OKT3). In our experimental application, we modified the protocol of the Miltenyi Biotec IFN-γ Secretion Assay - Detection Kit as illustrated in **Figure 1A**. The resulting optimised workflow, referred to hereafter as IFN-γ FlowCytoScreen, was developed for its application as a functional potency assay. First, we reduced the sample and volume amounts to minimise the number of DCs used for quality control testing and diverted from patient care. For this reason, we moved from tubes to the 96-well plate, which increased the number of samples that could be screened simultaneously and reduced operator time. Additionally, the preliminary data collected showed that the cytokine secretion time indicated on the data sheet was too short for this type of stimulation and that the results were not comparable to those obtained with ELISpot, in which stimulation and secretion occur O/N. Specifically, with a cytokine secretion time of 45 min we obtained percentages of IFN-γ secreting cells ranging from 3.2 to 5.5%, whereas with O/N stimulation we observed values ranging from 21.0 to 31.0% (3.8 to 9.6-fold higher). Based on these preliminary observations, O/N stimulation was selected for the assessment of IFN-γ production in the potency assay, thereby harmonising the stimulation conditions with those used for ELISpot. All samples were acquired using the gating strategy described in **Figure 1B** and all experiments included positive and no-DC controls consisting of DCs and T cells co-cultured with CytoStim and T cells co-cultured with OKT3 only, respectively (**Figure 1C**). For the positive control only, different CytoStim incubation times were evaluated to determine the optimal conditions. The highest IFN-γ production was observed after 2 or 3 h of CytoStim incubation; therefore, to streamline the procedure, a 2-h incubation time was selected as the preferred condition for the positive control (**Figure 1D**).

**Figure 1:**
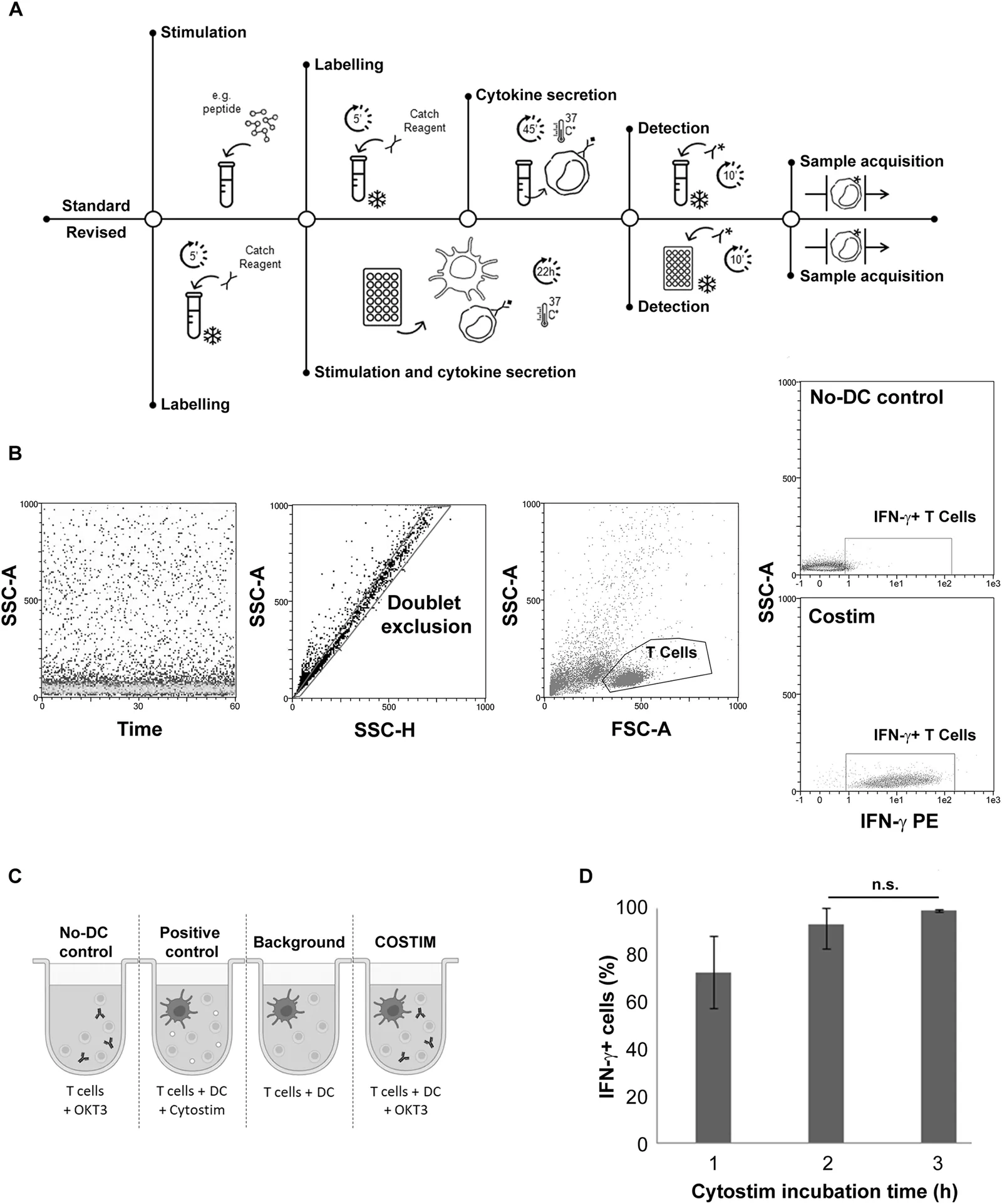
Method optimisation. **(A)** Graphical representation of the comparison between Miltenyi Biotec’s IFN-γ secretion assay protocol and our revised experimental workflow. **(B)** Representative flow cytometric dot plots showing the gating strategy used to identify the IFN-γ+ events. First, a dot plot was used with the parameter ‘Time’ versus SSC-A to remove event bursts caused by clogs or other transient problems during acquisition. Then, singlets were identified using SSC-A versus SSC-H to improve discrimination of highly granular T-DC heterodoublets. T cells were then gated using FSC-A versus SSC-A in linear scale, after which IFN-γ expression was assessed. The IFN-γ+ gate was defined using the no-DC control and applied consistently to all samples. **(C)** Seeding conditions of the revised protocol. **(D)** CytoStim incubation evaluated at 1, 2 and 3 h using three DC batches to determine optimal conditions. IFN-γ+ cell frequencies (%) are expressed as mean ± standard deviation (SD). n.s.: not significant.

### 3.2 Method validation

To determine whether the assay could specifically detect DC-induced stimulation, we co-cultured 1 x 10^5^ CD3+ cells with various concentrations of DCs or monocytes (**Figure 2A**). DCs consistently induced greater potency than monocytes, with the highest fold change (8.89) obtained with 2 x 10^4^ DCs compared to other dilutions (fold change range: 1.29-4.44). Therefore, a DC : CD3+ ratio of 1:5 was used in all subsequent experiments. For this defined ratio, the percentage variation in potency values obtained from DCs versus CD14+ cells ranged from 72.5% to 102.5%, meeting the acceptance criterion of >70% and demonstrating the specificity of the DC-induced biological response. As shown in **Figure 2B**, the addition of an Ab cocktail directed against the DC antigens with co-stimulatory activity decreased IFN-γ production, providing further evidence of specificity of the DC-induced stimulation (p=0.00002). The results obtained on three different DC batches met the established acceptance criteria; in fact the percentage of IFN-γ+ events in COSTIM+Abs was lower than the background percentage. According to the manufacturer’s instructions for the IFN-γ secretion assay, samples containing more than 5% IFN-γ secreting cells should be diluted to avoid non-specific staining. As the frequency of IFN-γ+ cells in our samples exceeded this threshold, we assessed the analytical specificity of cytokine detection without increasing the sample volume. To this end, catch reagent-labelled T lymphocytes were gradually replaced with cells from the acute lymphoblastic leukaemia REH cell line. These CD45+ cells could be labelled with the catch reagent, but they were unable to produce IFN-γ. Therefore, detection of IFN-γ positive REH cells would indicate non-specific cytokine capture. The DC:T lymphocyte ratio was maintained at 1:5 and a total of 1×10^5^ catch reagent-labelled cells were seeded in each well to maintain a constant cell density and therefore a comparable probability of cytokine capture by neighbouring cells. The potency of the T lymphocytes (CD3+) and REH cells (CD19+) was calculated for each sample (**Table 2**) and **Figure 2C** shows the histograms for the four conditions containing REH cells compared with the no-DC control. In the absence of REH cells, up to 6.5% of the CD19+ compartment scored positive for IFN-γ, representing the baseline level. After accounting for the baseline level, the IFN-γ+ REH fraction was only higher by 1.3% and 1.8% in the 60×10^3^ T + 40×10^3^ REH and 80×10^3^ T + 20×10^3^ REH conditions, respectively. Thus, the maximum estimated bystander contribution was 1.8% (**Figure 2D**). Moreover, there was a strong relationship between the number of T cells and T-cell potency (R^2^ = 0.98, p=0.001), whereas the relationship with REH potency was weaker (R^2^ = 0.64, p=0.10). The paired Cohen’s d of 1.05 indicated a large standardised difference between T and REH potency. To assess the method’s linearity with respect to the number of IFN-γ-producing cells, we seeded scalar concentrations of catch reagent-labeled T cells and DCs at a 1:5 ratio in the presence of CytoStim to obtain various quantities of IFN-γ-producing T cells. After 22 h of incubation, each sample was spiked-in with unlabeled T cells to reach 10^5^ CD3+ cells. This yielded a scalar quantity of IFN-γ+ cells among the samples, which were then detected and acquired. The results showed that R-squared (R^2^) was always ≥0.97 (acceptance criterion), indicating that the method is linear (**Figure 2E**). Similarly, we demonstrated accuracy in terms of recovery. Expected values were calculated by multiplying the percentage of DCs in culture and the measured value of IFN-γ+ cells obtained from 100% of the DCs (98.6%). The acceptance criterion for recovery was 80-120% (25), and we obtained an average recovery of 100.2% (**Table 3**). Furthermore, as shown in **Figure 2F**, there are no over- or underrated data in this assay compared to the expected values, indicating that the test is unaffected by systematic error. We assessed robustness by varying the incubation time (22 ± 1 h) or the OKT3 concentration (0.01 ± 0.002 µg/mL). In both cases, the CV was lower than the established acceptance criterion of 10% (0.1 and 0.3, respectively). The repeatability of the assay was demonstrated by comparing the potency obtained between replicates within the same culture plate from three independent experiments (CV ranging from 0.8 to 1.98%, the acceptance criterion is CV ≤ 10%, **Figure 2G**). Additionally, to determine the method’s reliability, we assessed the intermediate precision by analysing the inter-assay, inter-day, and inter-analyst variability in triplicate. In the first two cases, the acceptance threshold was set at CV ≤ 20%. CVs of 0.5% and 10.5%, respectively, for inter-assay and inter-day precision, indicated high reproducibility under several analytical conditions. Furthermore, as shown in **Figure 2H**, the potency data generated by the two analysts were highly consistent, yielding an ICC of 0.982 (the acceptance criterion is ICC > 0.5).

**Figure 2:**
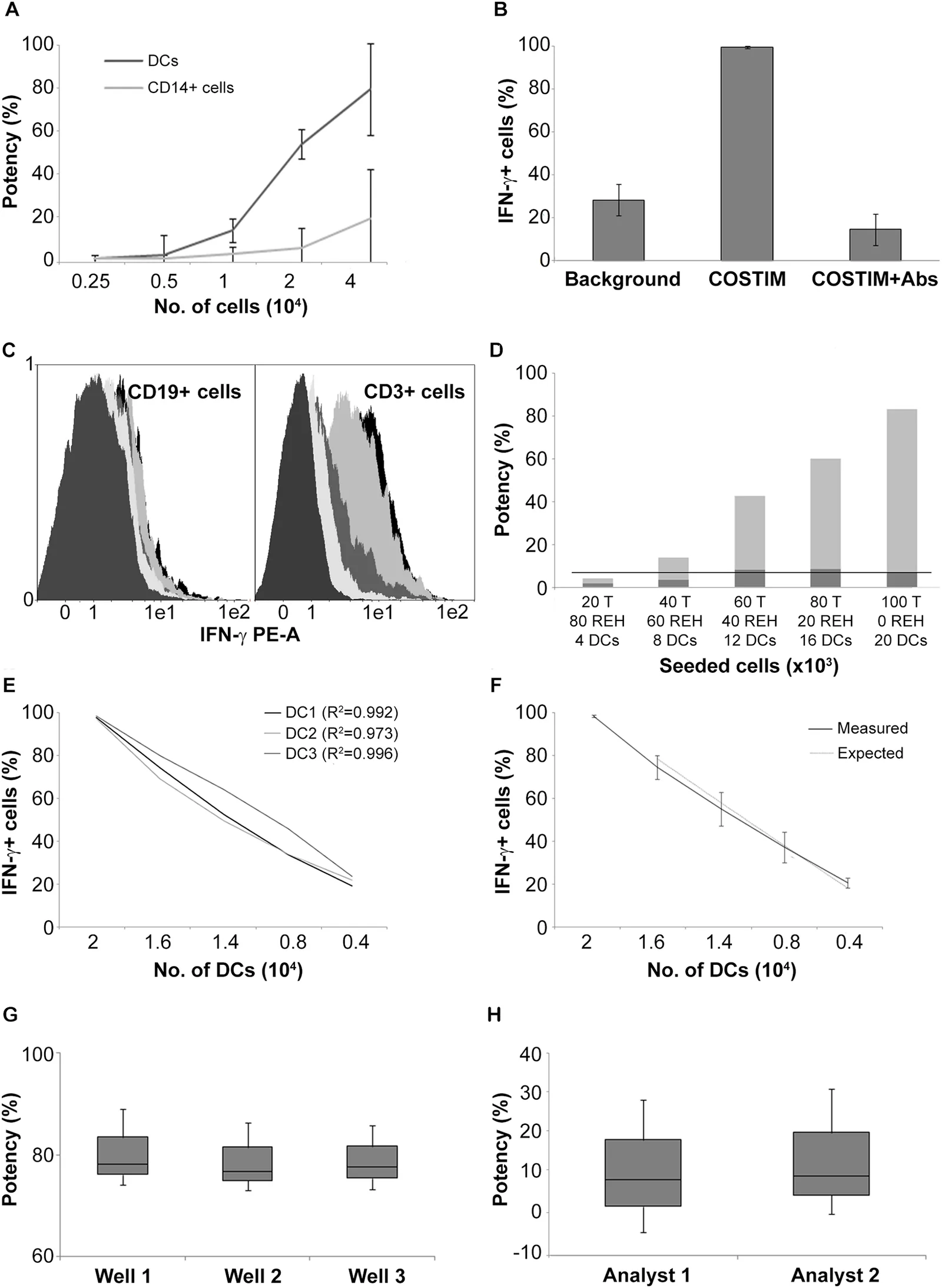
Method validation. **(A)** Potency value (mean ± SD) obtained from scalar dilutions of four batches of DC and monocytes. **(B)** IFN-γ+ cell frequencies (mean ± SD) with and without a cocktail of antibodies (anti-CD54, CD80, CD86) to determine the specific DC-induced stimulation. **(C)** Overlay of IFN-γ fluorescence intensity histograms for CD19+ (left) and CD3+ (right) cells. Histograms (front to back) correspond to: CTRL (no DCs), DCs with increasing T-cell/REH-cell ratios (20,000/80,000, 40,000/60,000, 60,000/40,000, and 80,000/20,000). **(D)** Potency values across different seeding conditions. Light grey bars represent T-cell-derived potency, while dark grey bars indicate the REH-associated contribution to the IFN-γ-positive signal. The horizontal line indicates the baseline level. **(E)** IFN-γ+ cell frequencies obtained by different dilutions of three DC batches. **(F)** Comparison between measured and calculated expected IFN-γ+ cells from three different experiments (mean ± SD). **(G)** Box plots showing intra-assay variability in potency across three wells in three independent experiments. **(H)** Box plots over three independent experiments illustrating the variability in potency between two analysts.

**Table 2:** Potency data obtained during non-specific binding experiments.

Seeded cells (10^3^)			Potency (%)	
T cells	REH cells	DCs	T cells	REH cells
20	80	4	2.3	1.7
40	60	8	10.1	3.3
60	40	12	33.5	7.8
80	20	16	49.8	8.3
100	0	20	74.1	6.5*

*Baseline level detected in the absence of REH cells and used for data correction.

**Table 3:** Accuracy and agreement between expected and measured values.

Percentage of DCs in culture (%)	100	80	60	40	20
Measured value	98.6	75.1	56.2	38.5	22.3
Expected value	–	78.9	59.2	39.4	19.7
Recovery	–	95.2	95.0	97.7	112.8
Recovery (mean)		100.2			

### 3.3 Comparative analysis of ELISpot and IFN-γ secretion assay

To assess the reliability of the developed method, we compared the modified IFN-γ secretion assay with the gold standard IFN-γ ELISpot assay (**Figure 3A**) while maintaining the previously described co-culture conditions. In particular, we co-cultured scalar dilutions of three different DC batches with a single batch of T cells. To evaluate the agreement between the two assays, we first compared the potency calculated using SFCs with ELISpot (**Figure 3B**) and the frequency of IFN-γ+ events (**Figure 3C**). However, no significant differences were observed between the three concentrations for two DC batches. This suggests that the percentage of IFN-γ+ events may not be sensitive enough to detect variations when the overall response is high. Nevertheless, the flow cytometric analysis revealed a shift in the fluorescence intensity distribution, indicating differential responses among the study cases, although the values of positive events remained consistently around 85% (**Figure 3D**). To further explore this observation, we calculated the iMFI and compared it with the SFC counts. The iMFI provided a more sensitive assessment of changes in cytokine production and exhibited the anticipated linear trend (**Figure 3E**). As shown in **Figure 3F**, a strong correlation was observed between iMFI and SFCs.

**Figure 3:**
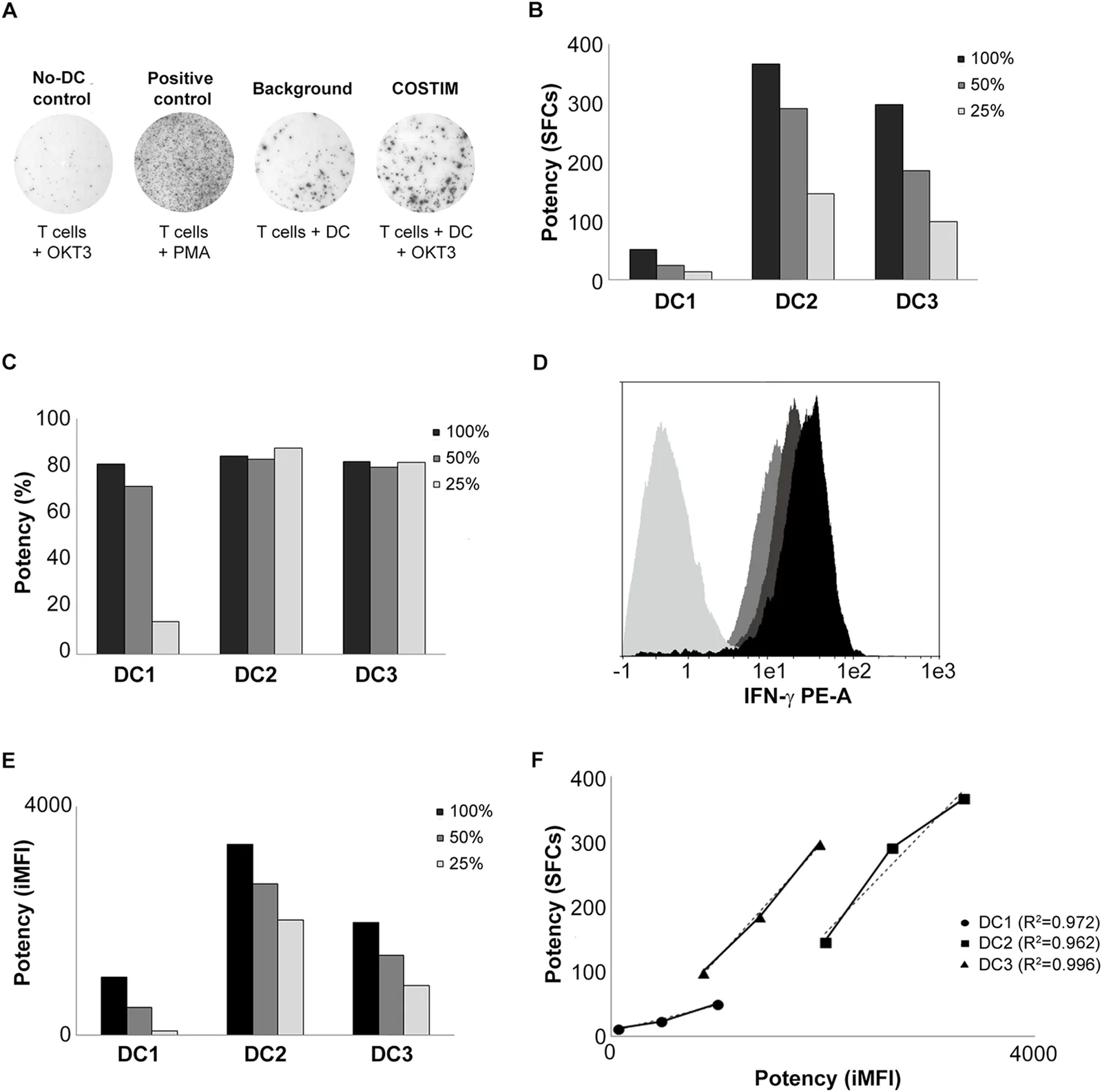
Comparative analysis of ELISpot and IFN-γ secretion assay. **(A)** IFN-γ ELISpot results from different seeding conditions. **(B)** Potency results, expressed in terms of SFCs, obtained by scalar dilutions of three DC batches using the ELISpot assay. **(C)** Potency results, expressed as frequency of IFN-γ+ cells, obtained with the our revised IFN-γ secretion assay under the same experimental conditions described previously. **(D)** The image shows the overlay of PE fluorescence intensity histograms of a representative sample. From left to right are the no-DC control and samples containing 25, 50, 100% DCs. **(E)** iMFI potency results from our revised assay under the same experimental conditions. **(F)** Line plot of data from three different DC batches tested with IFN-γ ELISpot and secretion assay.

After demonstrating the correlation between the assays, we expanded the ELISpot and IFN-γ secretion assays to include forty-five independent biological samples. We observed that IFN-γ FlowCytoScreen potency values ranged from −4.49% to 82.97%, with a median of 21.18% (interquantile range: 4.50–64.00%), indicating a broad distribution of potency values across the analysed samples and no consistent clustering of the assay response. To analyse the agreement between the two methods, we generated a Bland–Altman plot (**Figure 4A**). This plot indicates the absence of systematic error, as the mean difference between the two measurements (black line) lies within the 95% limits of agreement (gray lines). Additionally, the absence of a trend in the plotted data suggests that proportional error is not present. Finally, only four of the forty-five measurements fell outside the CI. During the analysis, 12 of the samples exhibited high spot density, making automated spot discrimination by the ELISpot reader more difficult (**Figure 4B**). These samples were included in the analysis of the forty-five paired measurements for the Bland–Altman plot, and the four values that fell outside the CI were among them.

**Figure 4:**
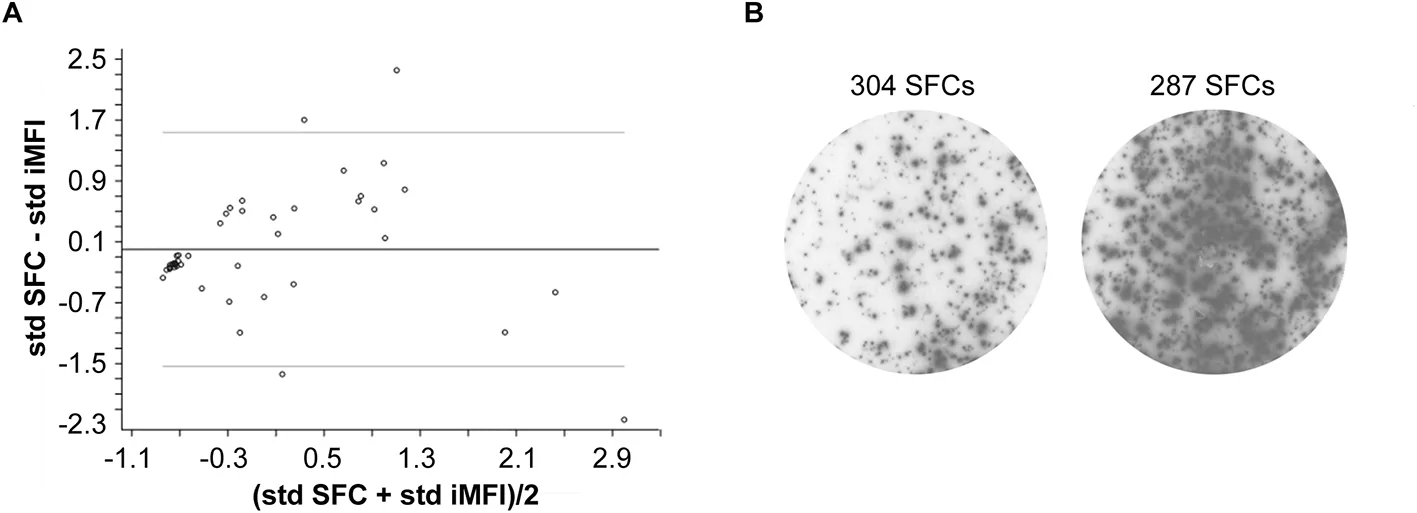
Results of the agreement between the methods. **(A)** Bland-Altman plot. The x-axis and the y-axis represent the mean and the difference between the two measurements, respectively. The black line represents the mean difference and the grey lines indicate the limits of agreement (mean difference ± 1.96 SD). **(B)** Representative ELISpot outputs. On the left an accurate SFC quantification, on the right an underestimated quantification due to a strong immune response.

To further investigate the statistical properties of the collected data, we calculated the ICC, which yielded a value of 0.82 (95% CI: 0.67-0.90), indicating good reliability between the two assays.

As detailed in **Table 4**, we also estimated the material costs per assay. Notably, we observed a 61% reduction in reagent and consumable costs when comparing ELISpot with IFNγ FlowCytoScreen. It is important to note that these estimates do not include the acquisition, depreciation, qualification, or maintenance costs of the flow cytometer or ELISpot reader. Additionally, the IFNγ FlowCytoScreen assay required approximately 6 h of laboratory work over 2 days, compared with 10 h over 4 days for the ELISpot assay. Therefore, IFNγ FlowCytoScreen reduced both material requirements and operator time compared with ELISpot.

**Table 4:** Cost assessment.

ELISpot				
Items	Cost	€/well	N°	€/assay
IFN-γ ELISpot assay kit	€ 465	€ 2.42	20	€ 48.4
Phorbol 12-myristate 13-acetate	€ 88.90	€ 0.009	4	€ 0.036
CellGenix^®^ GMP DC Medium	€ 208	€ 0.083	20	€ 1.66
OKT-3	€ 391	€ 0.24	8	€ 1.96
PVDF 96-well plate	€ 48	-	1	€ 48
Labor time (average per 96-well plate)	10 h over 4 days			
Total	€ 100			

IFNγ FlowCytoScreen				
Items	Cost	€/well	N°	€/assay
IFN-γ Secretion Assay – Detection Kits, human	€ 893	€ 8.93	4	€ 35.72
CytoStim™, human	€ 410	€ 1.64	1	€ 1.64
AIM-V Medium	€ 169	€ 0.07	4	€ 0.28
OKT-3	€ 391	€ 0.24	2	€ 0.48
96-well plate U bottom Cellstar^®^	€ 80	-	-	€ 0.8
BSA Stock solution	€ 332	€ 0.019	4	€ 0.074
AutoMACS Rinsing Solution	€ 175	€ 0.01	4	€ 0.04
Labor time (average per 96-well plate)	6 h over 2 days			
Total	€ 39			

### 3.4 Future direction: preliminary multiparametric characterisation of IFN-γ-producing cells

In addition to the validated IFN-γ readout, the IFN-γ FlowCytoScreen workflow was further explored for the multiparametric phenotypic characterisation of responding T-cell populations. These exploratory experiments combined our validated assay with a flow cytometry panel that included additional lineage and activation markers, enabling the identification of phenotypically defined cell subsets contributing to the IFN-γ response. The resulting data provided preliminary evidence that IFN-γ-producing cells could be characterised according to their phenotypic profile within the same experimental setting, as well as demonstrating heterogeneity in the phenotypic patterns of IFN-γ-producing cells among the analysed patient-derived cellular products (**Figure 5A, B**). As this application was not included in the analytical validation, further studies in a larger cohort will be required to validate these multiparametric measurements and to assess their potential utility for more detailed characterisation of the functional cellular response.

**Figure 5:**
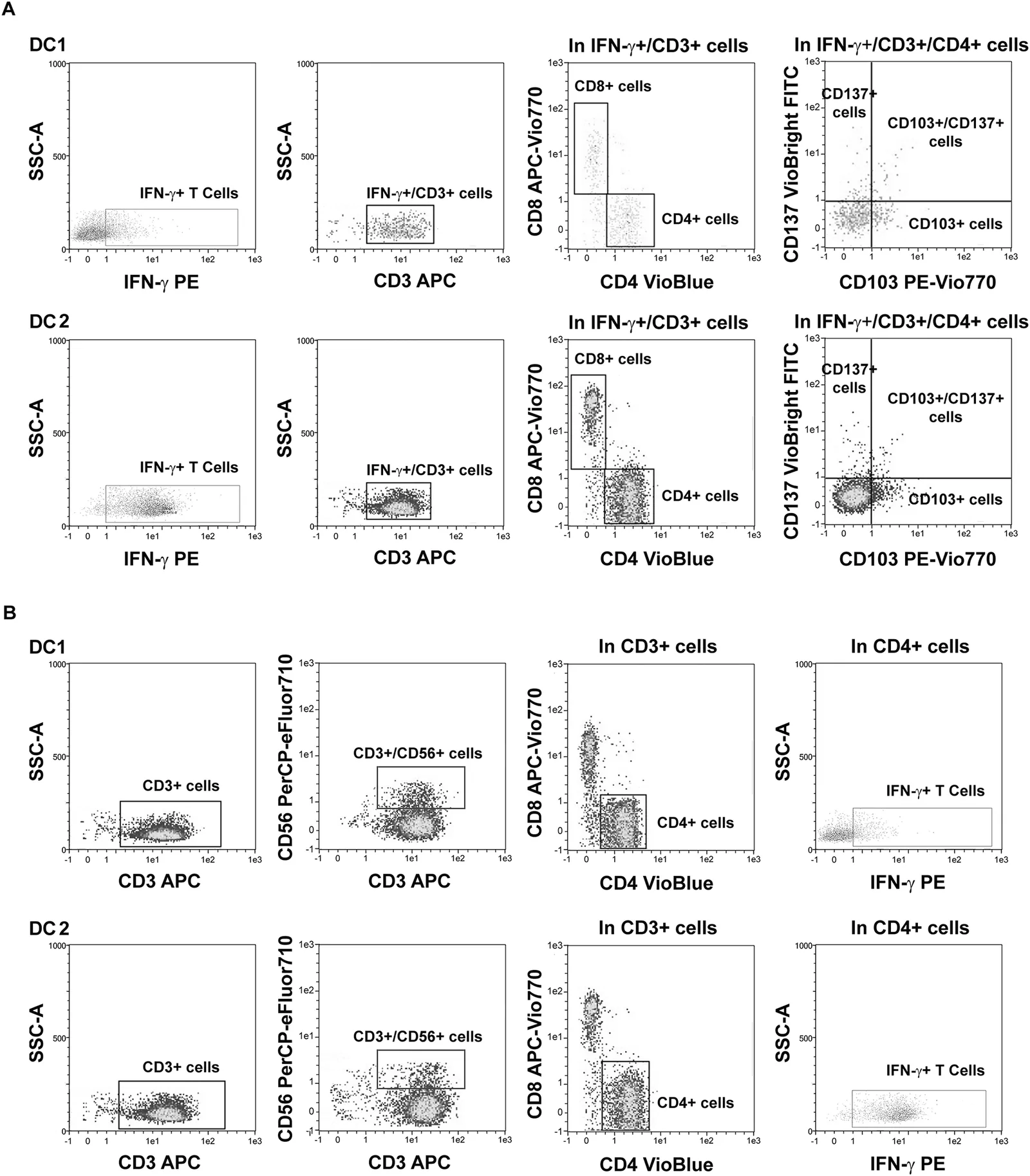
Complementary gating strategies for the phenotypic characterisation of IFN-γ-producing cells. The upstream gating steps were identical to those described in **Figure 1**. The data shown in both panels were obtained under the COSTIM condition of potency assays performed on two different cellular products. **(A)** Representative gating strategy starting from the IFN-γ^+^ population and then identifying the CD3^+^, CD4^+^ and CD8^+^ T-cell subsets. For example, the CD4^+^ population was characterised based on its expression of CD103 and CD137. **(B)** Representative gating strategy starting from the CD3^+^ population was used to identify CD56^+^ cells, as well as the CD4^+^ and CD8^+^ T-cell subsets. For example, the CD4^+^ population was analysed for IFN-γ expression.

## 4 Discussion

Reliable analytical tools and a quality control strategy are essential to ensuring that ATMPs meet the required safety and efficacy standards. This is especially important given the complexity and variability of these therapies. For this reason, potency assays are crucial to provide a comprehensive assessment of the functional attributes associated with the mechanism of action of these products and to ensure consistent evaluation of their biological activity (26–28). The analysis of cytokine secretion at the single-cell level is a powerful method for studying immune cell heterogeneity and function. Despite its limitations, the ELISpot is the most widely used assay to study protein secretion of individual cells (29). Standardisation and validation of the assay are difficult due to its inherent variability, labour-intensive nature, low scalability, challenges in the reliable quantification of high-frequency responses due to high spot density and multiplexing (9). Although alternative technologies for measuring cytokine production are available, they may differ in terms of assay format, readout and workflow. This highlights the need for validated and standardised approaches tailored to specific applications. For these reasons, our study has developed a novel and promising assay for assessing IFN-γ secretion, designed in accordance with current regulations and guidelines for validation (4, 5, 26). This ensures that the method used to test and monitor product characteristics (such as potency) is accurate and minimises the risk of error. Validation also helps to identify and correct any variables or sources of error that could affect the quality of the final product (30). In our study, we rigorously evaluated the assay for specificity, linearity, accuracy, robustness and precision to ensure its reliability and reproducibility. Additionally, multi-operator validation demonstrated that the method was not operator-dependent, which further supports its robustness and suitability for routine use. Since the gold standard is used for potency assessment within the DC vaccine stability program (31), it was necessary to compare the data generated by our new method with data obtained by the ELISpot assay. The reported results demonstrated a high degree of comparability between the two tests. The demonstrated correlation with ELISpot validates the reliability of IFN-γ FlowCytoScreen as a functional potency assay. Whether the magnitude of IFN-γ production measured in our potency assay is predictive of clinical benefit remains to be established and will be investigated in future studies. In addition to analytical comparability, the flow cytometry-based workflow offers several operational benefits, such as reduced processing time, greater standardisation, lower material requirements and more accessible results. It also improves operator safety by minimising the use of hazardous reagents and offers a more cost-effective workflow than ELISpot. Importantly, in our comparative analysis, IFN-γ FlowCytoScreen enabled the quantification of samples with very high frequencies of IFN-γ-secreting cells, for which reliable ELISpot spot enumeration was limited by high spot density. These findings suggest that IFN-γ FlowCytoScreen may provide a broader and more practical quantification range for high-frequency IFN-γ responses under the conditions tested. Moreover, flow cytometry is a powerful and versatile platform for high-dimensional analysis. It enables the simultaneous detection and quantification of multiple cytokines and additional cellular markers (32). Taken together, these considerations highlight the practical advantages of our IFN-γ FlowCytoScreen, establishing it as a valuable alternative for potency assessment in GMP laboratories and the pharmaceutical industry. The assay provides a standardised and efficient workflow, with reduced material requirements and processing time under the conditions evaluated. Finally, the proposed workflow could be readily adapted to quantify a broad range of cytokines, thereby expanding the resolution and scope of functional immune profiling. Beyond its use as a potency assay for DC-based vaccines, this approach has potential applications in immunomonitoring of patients enrolled in clinical trials, basic and translational immunology research, and the evaluation of immune responses to vaccination or immunotherapy, as well as in any setting requiring single-cell analysis of cytokine secretion. Following its validation as a potency assay for DC-based vaccines, the IFN-γ FlowCytoScreen is currently being explored in additional clinically relevant applications in our laboratories. In particular, it is being used for longitudinal immune monitoring of patients receiving DC-based vaccines, while a modified version of the workflow is under investigation as a potency assay for tumour-infiltrating lymphocyte-based therapies. These ongoing studies, together with future investigations extending the platform to additional cytokines and cell therapy products, will further define its utility as a versatile tool for functional immune monitoring and advanced therapy medicinal product (ATMP) characterisation. Overall, the IFN-γ FlowCytoScreen represents a validated, robust, and versatile platform for functional immune assessment, offering a standardised and scalable alternative to conventional cytokine release assays and supporting broader applications in translational immunology and cell therapy development.

## Data Availability

The raw data supporting the conclusions of this article will be made available by the authors, without undue reservation.
